# Age of initiation of cigarillo use among young adults: Findings from the Population Assessment of Tobacco and Health (PATH) study, 2013–2017

**DOI:** 10.1371/journal.pone.0264168

**Published:** 2022-03-31

**Authors:** Baojiang Chen, Kymberle L. Sterling, Meagan A. Bluestein, Elena Penedo, Arnold E. Kuk, Melissa B. Harrell, Cheryl L. Perry, Adriana Pérez

**Affiliations:** 1 Department of Biostatistics and Data Science, School of Public Health, The University of Texas Health Science Center at Houston (UTHealth), Austin Campus, Austin, Texas, United States of America; 2 Michael & Susan Dell Center for Healthy Living, School of Public Health, The University of Texas Health Science Center at Houston (UTHealth), Austin Campus, Austin, Texas, United States of America; 3 Department of Health Promotion and Behavioral Sciences, School of Public Health, The University of Texas Health Science Center at Houston (UTHealth), Dallas Campus, Dallas, Texas, United States of America; 4 Department of Epidemiology, Human Genetics and Environmental Sciences, School of Public Health in Austin Campus, The University of Texas Health Science Center at Houston (UTHealth), Austin, Texas, United States of America; University College London, UNITED KINGDOM

## Abstract

**Significance:**

Young adults, especially those who identify as racial/ethnic minorities, are legal targets of the tobacco industry. Cigarillo initiation is a risk among these vulnerable groups. Estimating the age of initiation of cigarillo use among young adults may inform the timing of prevention interventions.

**Methods:**

Weighted interval-censored survival analyses of the Population Assessment of Tobacco and Health (PATH) young adult (ages 18–24 at their first wave of adult participation) annual datasets were conducted (2013–2017). Young adult never cigarillo users (n = 7,101; represents N = 24,023,488) at their first wave of adult participation (2013–2016) were followed-up through 2014–2017 to estimate the age of initiation of ever, past 30-day and fairly regular cigarillo use outcomes. Differences by sex and by race/ethnicity, accounting for previous use of other tobacco products and marijuana and blunt use, were assessed using weighted interval-censored Cox proportional hazards models.

**Results:**

Among PATH young adults, by age 21, 5.8% initiated ever cigarillo use, 4.1% initiated past 30-day cigarillo use, and 1.4% initiated fairly regular cigarillo use. By age 26, 15% initiated ever cigarillo use, and 10.4% initiated past 30-day cigarillo use. Males had higher risk of initiating ever (AHR: 1.63, 95% CI: 1.37–1.95) and past 30-day cigarillo use (AHR: 1.65, 95% CI: 1.32–2.06) at earlier ages than females. Non-Hispanic Blacks had higher risk of initiating ever (AHR: 2.81, 95% CI: 2.26–3.50), past 30-day (AHR: 4.88, 95% CI: 2.95–5.09) and fairly regular cigarillo use (AHR: 4.62, 95% CI: 2.70–7.93) at earlier ages than non-Hispanic Whites. Hispanics had higher risk of initiating past 30-day cigarillo use at earlier ages than non-Hispanic Whites (AHR: 1.51, 95% CI: 1.12–2.03). Non-Hispanic Other race (i.e., Asian, multiracial, etc.) had lower risk of initiating ever (AHR: 0.43, 95% CI: 0.28–0.65) and past 30-day cigarillo use (AHR: 0.40, 95% CI: 0.26–0.63) at earlier ages than Non-Hispanic Whites.

**Conclusion:**

Along with those aged 21 and younger, interventions should target young adults over the age of 21, specifically males, non-Hispanic Black and Hispanic young adults, to stall initiation and progression of cigarillo use behaviors.

## Introduction

Cigars, a roll of combustible tobacco wrapped in a tobacco leaf, are responsible for several forms of cancer, lung disease and coronary heart disease [1]. These products contain similar toxic and carcinogenic compounds as cigarettes and are not a safe alternative to cigarette use [1]. Three major classes of cigar products are sold in the United States: traditional cigars (large cigars or “regular” cigars that are ≥ 7 inches and contain 5–20 grams of tobacco), cigarillos (smaller cigars that are bigger than a cigarette and contain 3 grams of tobacco), and filtered cigars (same size and shape of cigarettes, maintaining a filter and contain 1 gram of tobacco) [2].

Cigar sales data indicate that cigarillos are the most frequently used product among all the cigar product classes [3]. From 2012–2016 cigarillo sales increased by 78%, while sales of large cigars and filtered cigars decreased [3]. While cigar use has historically been a behavior associated with older adults, the popularity of cigarillo use has grown, particularly among young adults, males, non-Hispanic Blacks and those with previous tobacco product use experience [4–10]. The cigarillo industry’s marketing of appealing flavors, the affordability (e.g., often available for as little as $0.99), and accessibility of cigarillos (e.g., sold as a single stick or two for the price of one) often make these products attractive among price-sensitive young adults. Additionally, marijuana is often consumed in cigarillo products as blunts–or hollowed-out cigarillo products filled with marijuana [11–17]. The ability to use cigarillos as marijuana delivery devices may also be appealing to young adults [11–17].

After the Master Settlement Agreement banned direct and indirect tobacco product targeting and advertising to youth aged 17 and younger [18], the tobacco industry shifted their product marketing to young adults–legal targets who were considered “replacement” smokers [19]. A recent study examining data from the National Survey on Drug Use and Health indicated that the age of onset of cigarette use, has shifted from adolescence to young adults [20]. An analysis of cigar product use from the Population Assessment of Tobacco Use or Health (PATH) study, a longitudinal study of tobacco use and its effects on health in the United States, indicated that most cigar product initiation occurs during young adults [21]. Specifically, in 2013–2014, among PATH Wave 1 never cigar users, 12% reported initiating any cigar product use in the past 12 months, 7.2% initiated in the past 30-days, and 0.3% initiated frequent past 30-days use one year later [21]. In another PATH study, Kasza and colleagues (2020) found that young adults (18–24 year olds), being male, self-identifying as non-Hispanic Black and reporting ever use of cigarettes, ENDS, or hookah were significant correlates of higher odds of initiating cigar product use in the past 30-days in Waves 2–3 among adult never tobacco users at Wave 1 in 2013–2014 [22]. Other studies also found that reporting ever use of cigarette, ENDS, or marijuana were significantly associated with cigar product initiation [5, 7, 23–25].

The tobacco industry’s targeting of young adults, coupled with shifts in tobacco product use initiation patterns, highlight the importance of prevention efforts targeted toward this age group to prevent the onset and progression of cigarillo use behaviors and related health consequences. Discovering the precise age(s) when young adults initiate cigarillo use will help to educate the public and may inform intervention efforts to reduce the abuse liability of all cigar products. In this article, we present analyses from the first four waves of the PATH study that show the prospective distributions of age of initiation of cigarillo product use among young adults, aged 18–24, who reported never cigarillo use at their first wave of adult participation in the PATH study. Our study goes beyond prior work in its focus on cigarillo initiation by using data through wave 4 of PATH—the most frequently used product among all the cigar product classes [3]. In contrast to previous cigar use initiation studies, we prospectively estimate the age of cigarillo product initiation across three behavioral outcomes including ever use, past 30-day use, and fairly regular use. We estimated the age at which young adults initiated use for the overall sample, by sex, and by race/ethnicity, thus providing a fine-tuned estimate of the age that young adults initiate cigarillo use across these behavioral outcomes. We also investigated the difference of the age of initiation of different cigarillo use outcomes by previous use of other tobacco products (e.g., e-cigarette, cigarette, filtered cigar, traditional cigar, hookah, smokeless tobacco) and previous marijuana/blunt use. These data will inform the public domain, guide prevention interventions among young adults, and highlight the specific age, sex and race/ethnicity to target with communication and education campaigns for intervention.

## Methods

### Study design and participants

Secondary analyses of the PATH young adult data were conducted, where, we restrict the age range of participants at their first wave of adult participation between 18–24 years old. Four waves of PATH data were available to researchers at the time of analysis: wave 1: 2013–2014, wave 2: 2014–2015, wave 3: 2015–2016, wave 4: 2016–2017. In this study, participants who were 18–24 years old and had never used cigarillos at their first wave of adult participation in waves 1–3 (2013–2016) were included in the analysis. There were 4,405 participants who fit these criteria and entered the study at wave 1, 1,372 entered at wave 2 and 1,324 entered at wave 3. These participants had their outcomes followed-up from waves 2–4 (2014–2017). All participants aged 18 and older received and signed a written informed consent form from PATH before enrolling into the study. IRB approval for this study was obtained from the Committee for the Protection of Human Subjects at the University of Texas Health Science Center at Houston with number HSC-SPH-17-0368.

### Measures

In this study, we estimated the age of initiation of cigarillo use across three behavioral outcomes: (a) ever use; (b) past 30-day use; and (c) fairly regular use. For each outcome, participants who used cigarillos as intended/sold or as blunts (those who used cigarillos with marijuana inside) are included in the analysis [26, 27]. In each wave (waves 1–4), PATH asked all participants “Have you ever smoked a traditional cigar, cigarillo, or filtered cigar, even one or two puffs?” [26, 27]. Response options included “yes”, “no”, “don’t know” or “refused”. Among those who answered “yes”, questions for each product were asked, and the brand names and/or images for each specific product were also used to determine which product was used. PATH then derived a variable to differentiate between the different types of cigars, including cigarillos, to represent ever cigarillo use. Participants who reported “yes” to ever use of cigarillos were categorized as ever users of cigarillos. The “don’t know” and “refused” responses were classified as missing. The cross-sectional missing proportions for ever cigarillo use in the PATH study are 2.6%, 4.2%, and 4.4% in waves 2–4 [28]. In waves 2–4, PATH asked cigarillo users “In the past 30 days, on how many days did you smoke a cigarillo?” [26, 27]. PATH then derived a variable that assessed if participants had ever smoked cigarillos in the past 30 days (yes or no). The cross-sectional missing proportions for past 30-day cigarillo use in the PATH study are 0.1%, 0%, and 0% in waves 2–4 [28]. In waves 2–4, fairly regular use of cigarillos was measured by “Have you ever smoked cigarillos fairly regularly?” among cigarillo users. Response options included “yes”, “no”, “don’t know” or “refused”. The “don’t know” and “refused” responses were classified as missing. The cross-sectional missing proportions for fairly regular cigarillo use in the PATH study are 1.2%, 0%, and 0% in waves 2–4 [28].

### Other tobacco product

In addition to cigarillo use, PATH also measures ever use of other tobacco products (yes/no) using questions similar to cigarillos. We accounted for the effect of each other tobacco product on the age of initiation of cigarillo use. These tobacco products included cigarettes, e-cigarettes, traditional cigars, filtered cigars, hookah, and smokeless tobacco. We assessed participants’ other tobacco product use at the wave prior to each cigarillo initiation outcome.

### Blunt and marijuana use

In wave 1, PATH asked participants who have seen or heard of a traditional cigar, cigarillo or filtered cigar and can identify which type of cigar they have heard of (1) “Have you ever smoked part or all of a cigar, cigarillo or filtered cigar with marijuana in it (blunt)?”. In waves 2–4, PATH asked all adult participants (2) “In the past 12 months, have you smoked part or all of a traditional cigar, cigarillo or filtered cigar with marijuana in it?”. Response options for both questions included “yes”, “no”, “don’t know” or “refused”. The “don’t know” and “refused” responses were classified as missing. In wave 1, PATH asked all adult participants who did not already report using blunts (3) “Have you ever used marijuana, hash, THC, grass, pot, or weed?”. In waves 2–4, PATH also asked (4) “In the past 12 months, have you used marijuana, hash, THC, grass, pot or weed?” among adult respondents who did not report blunt use. Response options for both questions (3) and (4) included “yes”, “no”, “don’t know” or “refused”. The “don’t know” and “refused” responses were classified as missing. For those who answered “yes” for question (1) or (2) only, we characterized them as blunt users, and for those who answered “yes” for question (3) or (4), we characterized them as marijuana users. For those who answered “no” to all questions, we characterized them as never blunt or marijuana users.

### Sex and race/ethnicity

Sex was classified as males or females. This variable was imputed using the household information by PATH in 2013–2014. In PATH, race was assessed as White race alone, Black race alone, Asian race alone, and other race (including multi-racial), and ethnicity was categorized as either Hispanic or Non-Hispanic. To be comparable to those in prior Surgeon General’s reports [29, 30] and other studies, we classified race/ethnicity into four categories: Non-Hispanic White, Hispanic, Non-Hispanic Black, Non-Hispanic Other (Asian, multi-race, and other races).

### Interval-censored age of initiation of cigarillos

The exact date of initiation of each cigarillo use outcome (ever, past 30-day, and fairly regular use) was not available in PATH, as it is not feasible to ask participants the exact date that they initiated cigarillo use. Participants’ birthdays are also not included in the restricted-use dataset. We used 2 variables: (i) the participant’s age (in years) at the first wave of PATH adult participation and (ii) the number of weeks between survey waves to estimate a lower and upper age bound for each cigarillo use outcome. For all participants, the lower bound was the age at the last wave where the subject reported non-use of each cigarillo use outcome. For those who became users, the upper bound was the lower bound age plus the number of weeks between survey waves that the participant first reported initiation of each cigarillo outcome. For those who remained non-users, the upper age bound was censored. For example, if a subject was 18 years old at wave 1, with 40 weeks as the number of weeks between wave 1 and wave 2, then the age at wave 2 was 18+(40/52) = 18.77 years; if the number of weeks between wave 2 and wave 3 was 60 weeks, then the age at wave 3 was 18+(40+60)/52 = 19.92 years. Suppose this subject reported never cigarillo use at waves 1 and 2, but ever cigarillo use at wave 3, the age interval of ever cigarillo use was (18.77, 19.92].

### Statistical analysis

To account for the complex survey design, all analyses incorporated sampling weights and the 100 balance repeated replicate weights with Fay’s factor of 0.3 [31]. Weighted means and standard errors for continuous variables, and weighted frequencies and percentages for categorical variables are reported. The age of initiation of ever, past 30-day and fairly regular cigarillo use was estimated using non-parametric survival analyses for interval-censored data [32, 33]. The hazard functions and its 95% confidence intervals (CIs) were estimated overall, as well stratified by sex and race/ethnicity, and the hazard functions were also plotted. Differences in the age of initiation of the different cigarillo use outcomes (i.e., ever use, past 30-day use, fairly regular use) by sex, by race/ethnicity, as well as controlling for the effect of previous use of other tobacco products and previous marijuana/blunt use were conducted using interval-censored Cox proportional hazards models with the piecewise constant baseline hazard function [34]. Separate hazard functions showing the full distributions of the ages of initiation and their 95% CIs are also reported for the cigarillo use behaviors with significant effects by sex or by race/ethnicity. All statistical analyses were completed in SAS version 9.4 using the Inter-university Consortium for Political and Social Research server hosted by the University of Michigan.

## Results

As shown in Table 1, there were n = 7,101 young adults, representing N = 24,023,488 participants who had never used cigarillos at their first wave of adult PATH participation (2013–2016). Among these participants, almost 75% entered PATH at wave 1 (2013–2014), 56% were female, 22% were Hispanic, 53% were Non-Hispanic White, 13% were Non-Hispanic Black, and 13% were Non-Hispanic other race/ethnicity. Their mean age at their first wave of adult participation was about 20.2 years old.

**Table 1:** Sociodemographic characteristics of PATH USA young adults (aged 18–24 at the first wave of adult participation) never cigarillo users at their first wave of adult participation 2013–2016 (n = 7,101, N = 24,023,488)*.

Variables	Unweighted Frequency	Weighted Frequency (%)	Weighted SE
**Wave of Entry Into PATH**		** **	
Wave 1	4,405	18,118,502 (75.4)	0.32
Wave 2	1,372	3,039,685 (12.7)	0.24
Wave 3	1,324	2,865,300 (11.9)	0.22
**Sex**		** **	
Female	4,084	13,546,385 (56.4)	0.45
Male	3,013	10,465,493 (43.6)	0.45
Missing	4	11,610	
**Race/ethnicity**		** **	
Hispanic	1,926	5,269,301 (22.0)	0.63
Non-Hispanic White	3,471	12,679,347 (52.9)	1.00
Non-Hispanic Black	990	3,042,329 (12.7)	0.49
Non-Hispanic Other^Ϯ^	694	2,987,353 (12.5)	0.84
Missing	20	45,158	
**Age at entry into study (mean, SE)**	7,101	20.2 (0.04)	0.04

*PATH Restricted file received disclosure to publish: July 15, 2020, October 15, 2020. United States Department of Health and Human Services. National Institutes of Health. National Institute on Drug Abuse, and United States Department of Health and Human Services. Food and Drug Administration. Center for Tobacco Products. Population Assessment of Tobacco and Health (PATH) Study [United States] Restricted-Use Files. ICPSR 36231-v13. Ann Arbor, MI: Inter-university Consortium for Political and Social Research [distributor], November 5, 2019. Https://doi.org/10.3886/ICPSR36231.v23.

^Ϯ^Non-Hispanic Others include Asian, multi-race, etc.

As shown in Table 2, prior to ever cigarillo initiation, 9.1% had ever used other cigars as blunts, 10.3% had ever used marijuana, 32.8% had ever used cigarettes, 28.9% had ever used e-cigarettes, 8.7% had ever used traditional cigar, 4.6% had ever used filtered cigar, 30% had ever used hookah, and 5.3% had ever used smokeless tobacco products. Findings on previous use of other tobacco products, blunts, and marijuana prior to initiation of past 30-day and fairly regular use are also shown in Table 2.

**Table 2:** Previous use of other tobacco products, blunt, and marijuana use at the wave prior to initiation of each cigarillo outcome.

Variable	Ever cigarillo use		Past 30-day cigarillo use		Fairly regular cigarillo use	
	n^a^	N^b^ (%)	n^a^	N^b^ (%)	n^a^	N^b^ (%)
Blunt use	823	2,191,067 (9.1)	875	2,342,845 (9.8)	970	2,629,137 (10.9)
Marijuana use	799	2,470,321 (10.3)	785	2,445,262 (10.2)	764	2,387,352 (9.9)
Never blunt or marijuana use	5,478	19,360,051 (80.6)	5,441	19,235,381 (80.0)	5,367	19,006,998 (79.1)
Ever cigarette use	2,700	7,872,153 (32.8)	2,759	8,072,939 (34.5)	2,843	8,351,150 (35.7)
Ever e-cigarette use	2,522	6,927,286 (28.9)	2,593	7,164,969 (30.9)	2,708	7,526,912 (32.5)
Ever traditional cigar use	661	2,074,112 (8.7)	746	2,338,922 (10.2)	885	2,731,984 (11.9)
Ever filtered cigar use	403	1,100,368 (4.6)	439	1,182,559 (5.1)	555	1,490,283 (6.5)
Ever hookah use	2,512	7,196,241 (30.0)	2,559	7,371,134 (31.6)	2,632	7,632,333 (32.8)
Ever smokeless use	455	1,265,448 (5.3)	471	1,321,029 (5.8)	491	1,380,872 (6.0)

*PATH Restricted file received disclosure to publish: June 22, 2021, June 25, 2021. United States Department of Health and Human Services. National Institutes of Health. National Institute on Drug Abuse, and United States Department of Health and Human Services. Food and Drug Administration. Center for Tobacco Products. Population Assessment of Tobacco and Health (PATH) Study [United States] Restricted-Use Files. ICPSR 36231-v13. Ann Arbor, MI: Inter-university Consortium for Political and Social Research [distributor], November 5, 2019. Https://doi.org/10.3886/ICPSR36231.v23.

a: n represents unweighted sample frequency

b: N represents weighted frequency.

**Fig 1:**
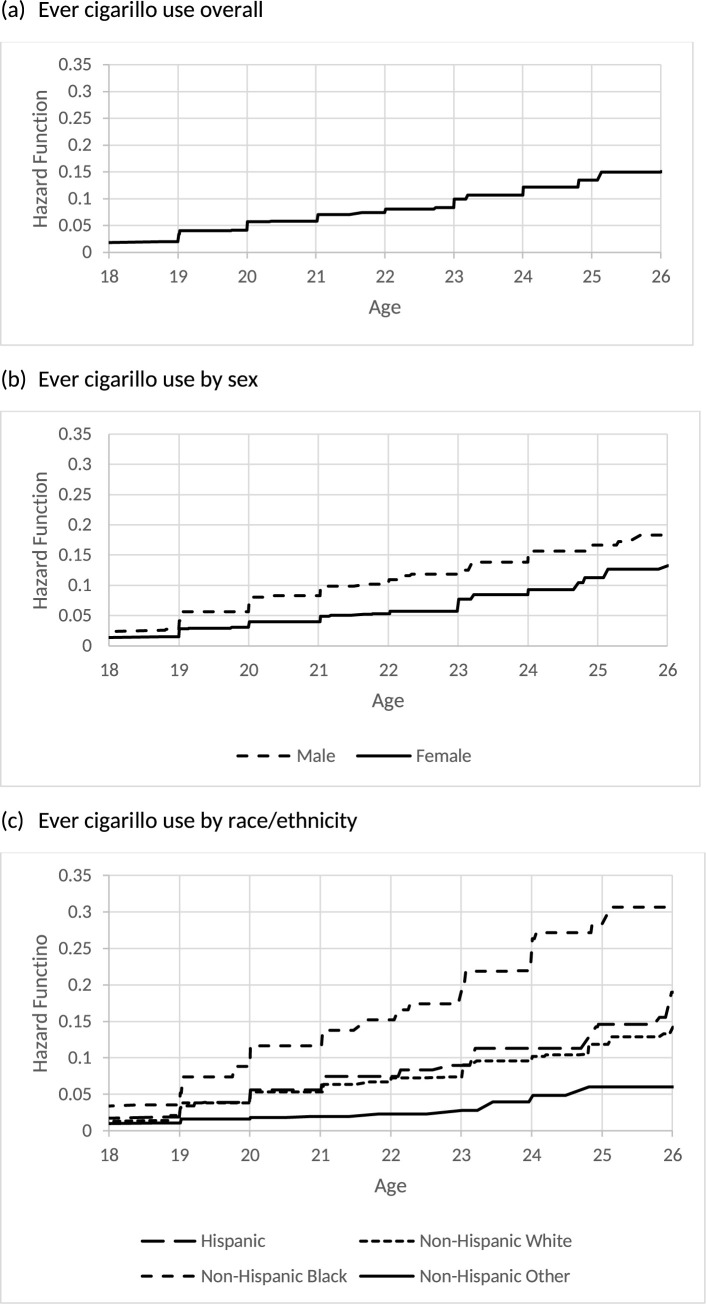
Estimated hazard function for age of initiation of ever cigarillo use.

**Fig 2:**
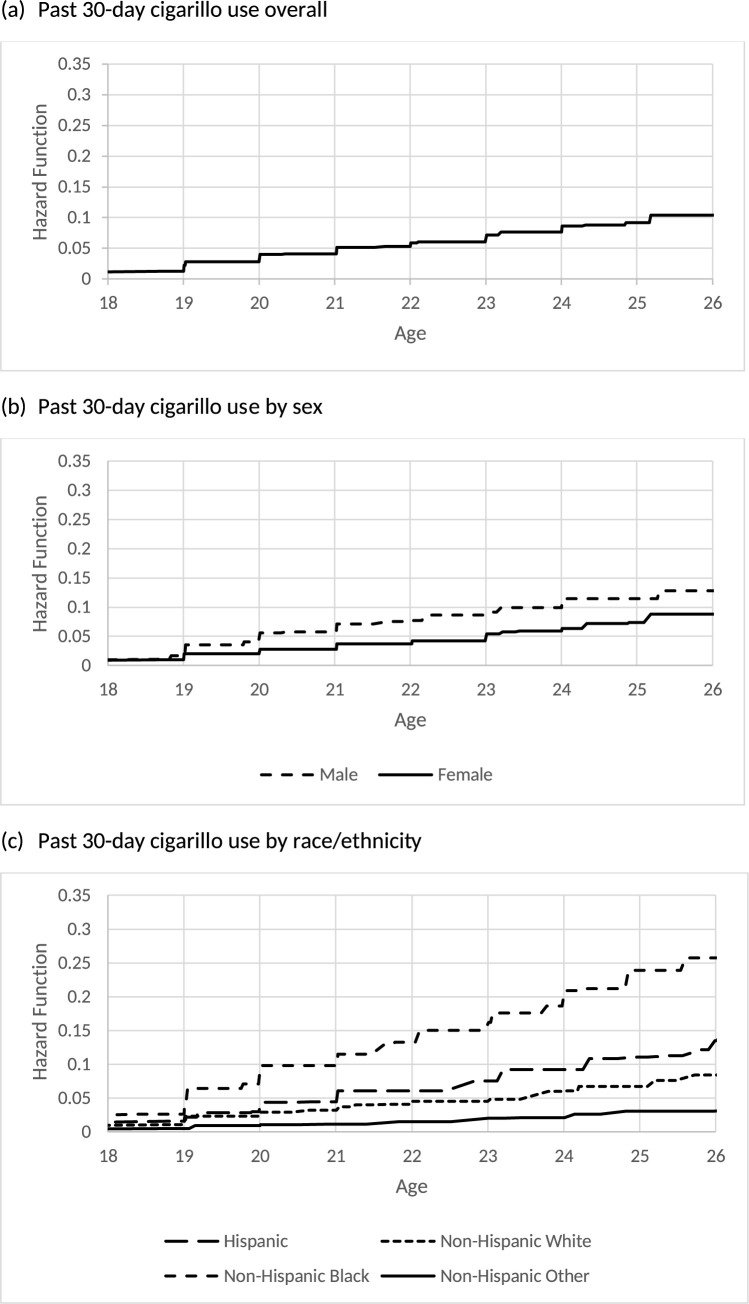
Estimated hazard function for age of initiation of past 30-day cigarillo use.

**Fig 3:**
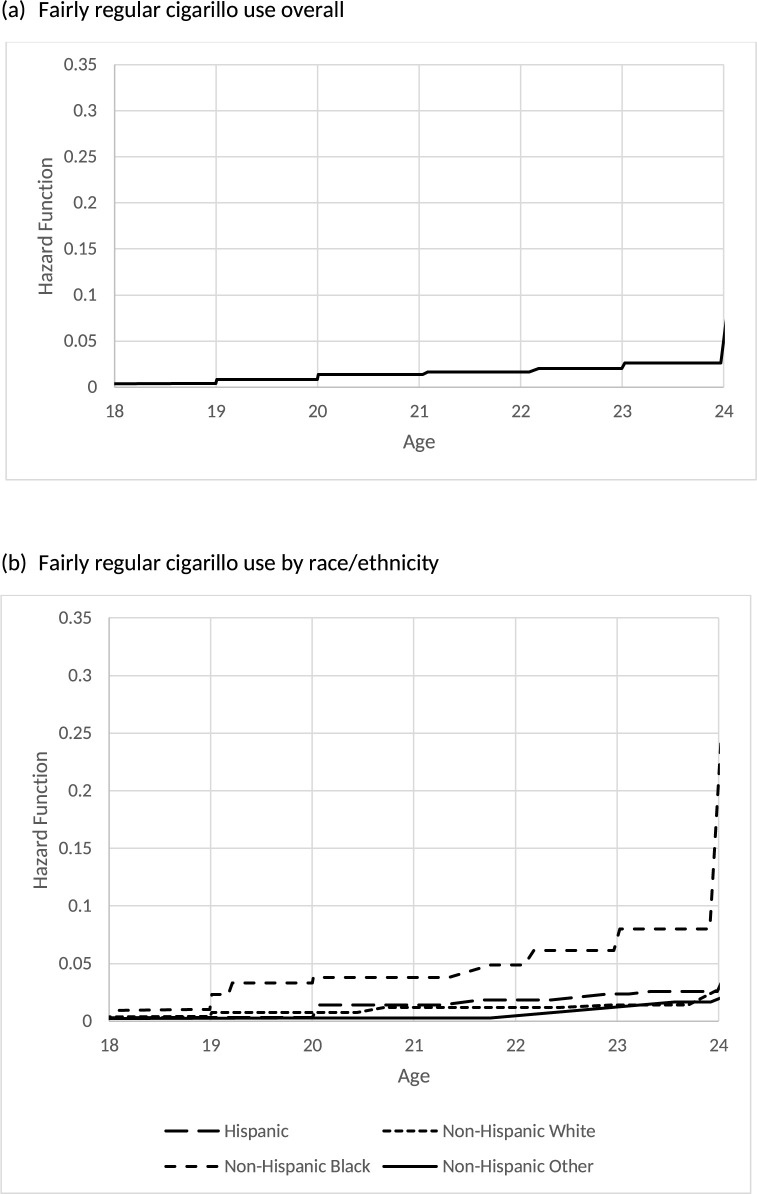
Estimated hazard function for age of initiation of fairly regular cigarillo use.

Table 3 reports the estimated hazard functions and its 95% CIs of the age of initiation of each cigarillo use outcome for the overall sample of young adults. By 21 years old, 5.8% of young adults initiated ever cigarillo use, 4.1% initiated past 30-day cigarillo use, and 1.4% initiated fairly regular cigarillo use. By 26 years old, 15% of young adults initiated ever cigarillo use and 10.4% initiated past 30-day cigarillo use. Figs 1A, 2A and 3A also display the overall hazard functions of the ages of initiation for each outcome.

**Table 3:** Estimated hazard functions^a^ (and 95% confidence intervals)^b^ of the age of initiation of each cigarillo use outcome for the overall sample of PATH* young adults (18–24 at the first wave of adult participation).

Age	Ever Use (%)	Past 30-Day Use (%)	Fairly Regular Use (%)
**18**	0.0	0.0	0.0
**19**	2.0 (1.7–2.4)	1.3 (1.0–1.6)	0.4 (0.2–0.6)
**20**	4.2 (3.6–4.7)	2.8 (2.3–3.3)	0.8 (0.6–1.1)
**21**	5.8 (5.2–6.4)	4.1 (3.5–4.6)	1.4 (1.0–1.7)
**22**	7.4 (6.6–8.2)	5.3 (4.7–6.0)	1.7 (1.3–2.1)
**23**	8.3 (7.4–9.2)	6.0 (5.3–6.8)	2.0 (1.5–2.6)
**24**	10.7 (9.5–12.0)	7.6 (6.6–8.6)	2.6 (1.9–3.3)
**25**	13.5 (11.8–15.2)	9.2 (8.1–10.3)	NA
**26**	15.0 (13.1–16.9)	10.4 (8.9–12.0)	NA

^a^: Hazards are reported as cumulative percentages (i.e., cumulative incidence), which do not adjust for other covariates.

^b^: 95% CI: Turnbull 95% confidence interval.

*PATH Restricted file received disclosure to publish: July 15, 2020, October 15, 2020. United States Department of Health and Human Services. National Institutes of Health. National Institute on Drug Abuse, and United States Department of Health and Human Services. Food and Drug Administration. Center for Tobacco Products. Population Assessment of Tobacco and Health (PATH) Study [United States] Restricted-Use Files. ICPSR 36231-v13. Ann Arbor, MI: Inter-university Consortium for Political and Social Research [distributor], November 5, 2019. Https://doi.org/10.3886/ICPSR36231.v23.

NA: There is not enough sample size to provide stable estimates at that age.

Table 4 reports the crude and adjusted hazard ratios and 95% CIs exploring the differences in the age of initiation for each cigarillo outcome for sex, race/ethnicity, previous use of marijuana or blunts, and previous use of other tobacco products. After adjusting for covariates, males were 63% (HR: 1.63, 95% CI: 1.37–1.95) more likely to initiate ever cigarillo use and were 65% (HR: 1.65, 95% CI: 1.32–2.06) more likely to initiate past 30-day cigarillo use at younger ages than females. Non-Hispanic Black young adults were 181% more likely (HR: 2.81, 95% CI: 2.26–3.50) to initiate ever cigarillo use, were 388% more likely (HR: 4.88, 95% CI: 2.95–5.09) to initiate past 30-day cigarillo use, and were 362% (HR: 4.62, 95% CI: 2.70–7.93) more likely to initiate fairly regular cigarillo use at earlier ages than Non-Hispanic White young adults. Non-Hispanic Other race/ethnicity were 57% less likely (HR: 0.43, 95% CI: 0.28–0.65) to initiate ever cigarillo use and were 60% less likely (HR: 0.40, 95% CI: 0.26–0.63) to initiate past 30-day cigarillo use at earlier ages than Non-Hispanic White young adults. Hispanic young adults were 51% more likely (HR: 1.51, 95% CI: 1.12–2.03) to initiate past 30-day cigarillo use at earlier ages than Non-Hispanic White young adults. Compared to never blunt or marijuana users, young adults who reported previous blunts use were 194% more likely (HR: 2.94, 95% CI: 2.31–3.73) to initiate ever cigarillo use, were 238% more likely (HR: 3.38, 95% CI: 2.53–4.53) to initiate past 30-day cigarillo use, and were 250% more likely (HR: 3.50, 95% CI: 1.92–6.40) to initiate fairly regular cigarillo use at earlier ages. Compared to never blunt or marijuana users, young adults who reported previous marijuana use were 79% more likely (HR: 1.79, 95% CI: 1.40–2.28) to initiate ever cigarillo use, were 103% more likely (HR: 2.03, 95% CI: 1.53–2.69) to initiate past 30-day cigarillo use, and were 288% more likely (HR: 3.88, 95% CI: 1.95–7.71) to initiate fairly regular cigarillo use at earlier ages. Young adults who reported previous cigarettes use were 32% more likely (HR: 1.32, 95% CI: 1.05–1.66) to initiate ever cigarillo use, were 53% more likely (HR: 1.53, 95% CI: 1.20–1.94) to initiate past 30-day cigarillo use at earlier ages. Young adults who reported previous filtered cigars use were 179% more likely (HR: 2.79, 95% CI: 1.49–5.24) to initiate fairly regular cigarillo use at earlier ages. Young adults who reported previous hookah use were 39% less likely (HR: 0.61, 95% CI: 0.38–0.97) to initiate fairly regular cigarillo use at earlier ages. Young adults who reported previous smokeless tobacco products use were 45% more likely (HR: 1.45, 95% CI: 1.02–2.05) to initiate ever cigarillo use, and were 83% more likely (HR: 1.83, 95% CI: 1.23–2.71) to initiate past 30-day cigarillo use at earlier ages.

**Table 4:** Hazard ratios (and 95% confidence intervals) for each cigarillo outcome by sex, race/ethnicity, and previous use of other tobacco products, blunt or marijuana use*.

Variable	Ever Use	Past 30-Day Use	Fairly Regular Use
**Univariate Analysis**			
**Sex**			
Female	1.00	1.00	1.00
Male	**1.72 (1.47–2.04)**	**1.75 (1.41–2.13)**	1.45 (0.97–2.13)
**Race**			
Non-Hispanic White	1.00	1.00	1.00
Non-Hispanic Black	**2.46 (1.95–3.11)**	**3.34 (2.53–4.41)**	**4.28 (2.61–7.03)**
Non-Hispanic Other^Ϯ^	**0.40 (0.26–0.62)**	**0.36 (0.23–0.58)**	0.35 (0.09–1.34)
Hispanic	1.16 (0.90–1.48)	**1.52 (1.14–2.02)**	1.33 (0.74–2.42)
**Previous blunt or marijuana use**			
Never use	1.00	1.00	1.00
Blunt use	**3.96 (3.18–4.93)**	**4.57 (3.55–5.89)**	**4.80 (3.09–7.48)**
Marijuana use	**1.85 (1.48–2.31)**	**1.97 (1.52–2.55)**	**3.11 (1.70–5.70)**
**Previous cigarette use**	**1.74 (1.42–2.12)**	**1.80 (1.46–2.22)**	**1.93 (1.25–2.96)**
**Previous E-cigarette use**	**1.82 (1.50–2.20)**	**1.65 (1.33–2.05)**	**1.50 (1.04–2.16)**
**Previous traditional cigar use**	**1.75 (1.34–2.29)**	**1.48 (1.11–1.99)**	**2.08 (1.33–3.25)**
**Previous filtered cigar use**	**2.10 (1.60–2.75)**	**1.73 (1.26–2.38)**	**4.46 (2.72–7.31)**
**Previous hookah use**	**1.36 (1.16–1.61)**	**1.32 (1.08–1.61)**	1.25 (0.85–1.82)
**Previous smokeless use**	**2.29 (1.69–3.11)**	**2.39 (1.71–3.34)**	1.02 (0.46–2.26)
**Multivariable Analysis**			
**Sex**			
Female	1.00	1.00	1.00
Male	**1.63 (1.37–1.95)**	**1.65 (1.32–2.06)**	1.48 (0.94–2.35)
**Race**			
Non-Hispanic White	1.00	**1.00**	1.00
Non-Hispanic Black	**2.81 (2.26–3.50)**	**4.88 (2.95–5.09)**	**4.62 (2.70–7.93)**
Non-Hispanic Other^Ϯ^	**0.43 (0.28–0.65)**	**0.40 (0.26–0.63)**	0.36 (0.09–1.42)
Hispanic	1.13 (0.88–1.44)	**1.51 (1.12–2.03)**	1.24 (0.65–2.36)
**Previous blunt or marijuana use**			
Never use	1.00	1.00	1.00
Blunt use	**2.94 (2.31–3.73)**	**3.38 (2.53–4.53)**	**3.50 (1.92–6.40)**
Marijuana use	**1.79 (1.40–2.28)**	**2.03 (1.53–2.69)**	**3.88 (1.95–7.71)**
**Previous cigarette use**	**1.32 (1.05–1.66)**	**1.53 (1.20–1.94)**	1.54 (0.89–2.68)
**Previous E-cigarette use**	1.24 (0.97–1.59)	1.06 (0.80–1.41)	0.89 (0.53–1.50)
**Previous traditional cigar use**	1.17 (0.87–1.56)	1.03 (0.76–1.40)	1.27 (0.74–2.19)
**Previous filtered cigar use**	1.33 (0.97–1.83)	1.06 (0.74–1.51)	**2.79 (1.49–5.24)**
**Previous hookah use**	0.84 (0.71–1.01)	0.84 (0.69–1.04)	**0.61 (0.38–0.97)**
**Previous smokeless use**	**1.45 (1.02–2.05)**	**1.83 (1.23–2.71)**	0.69 (0.29–1.68)

*PATH Restricted file received disclosure to publish: July 15, 2020, October 15, 2020, June 22, 2021, June 25, 2021. United States Department of Health and Human Services. National Institutes of Health. National Institute on Drug Abuse, and United States Department of Health and Human Services. Food and Drug Administration. Center for Tobacco Products. Population Assessment of Tobacco and Health (PATH) Study [United States] Restricted-Use Files. ICPSR 36231-v13. Ann Arbor, MI: Inter-university Consortium for Political and Social Research [distributor], November 5, 2019. Https://doi.org/10.3886/ICPSR36231.v23.

ϮNon-Hispanic Others include Asian, multi-race, etc.

Table 5 reports the estimated hazards and its 95% CIs of the age of initiation of ever and past 30-day cigarillo use by sex. Males exhibited a heightened risk for initiating ever use at earlier ages compared to females. For example, by age 21 8.2% of males and 3.9% of females initiated ever cigarillo use. By age 21, 5.8% of males and 2.8% of females initiated past 30-day cigarillo use. By age 26, 18.3% of males and 12.7% of females initiated ever cigarillo use. By age 26, 12.8% of males and 8.8% of females initiated past 30-day cigarillo use. Figs 1B and 2B also display the hazard functions of the ages of initiation for these two outcomes stratified by sex. There were no statistically significant differences in the age of initiation of fairly regular cigarillo use by sex.

**Table 5:** Estimated hazards^a^ (and 95% confidence intervals)^b^ of the age of initiation of each cigarillo use outcome for the overall sample of PATH USA young adult (aged 18–24 at the first wave of participation) by sex*.

Age	Ever use		Past 30-day use	
	Males	Females	Males	Females
**18**	0	0	0	0
**19**	2.8 (2.1–3.5)	1.5 (1.1–1.9)	1.7 (1.2–2.2)	1.0 (0.7–1.3)
**20**	5.6 (4.6–6.6)	3.1 (2.5–3.6)	4.0 (3.1–5.0)	2.0 (1.6–2.5)
**21**	8.2 (7.2–9.3)	3.9 (3.3–4.6)	5.8 (4.8–6.7)	2.8 (2.2–3.3)
**22**	10.2 (8.7–11.7)	5.3 (4.4–6.2)	7.5 (6.2–8.8)	3.7 (2.9–4.5)
**23**	11.8 (10.2–13.5)	5.7 (4.8–6.7)	8.6 (7.2–10.0)	4.3 (3.3–5.2)
**24**	13.9 (12.1–15.6)	8.5 (6.9–10.0)	9.9 (8.3–11.5)	6.0 (4.7–7.2)
**25**	16.7 (14.5–18.8)	11.3 (9.3–13.3)	11.4 (9.7–13.2)	7.4 (6.0–8.8)
**26**	18.3 (15.6–21.0)	12.7 (10.5–14.9)	12.8 (10.5–15.1)	8.8 (7.0–10.7)

^a^: Hazards are reported as cumulative percentages (i.e., cumulative incidence), which do not adjust for other covariates.

^b^: 95% CI: Turnbull 95% confidence interval.

*PATH Restricted file received disclosure to publish: July 15, 2020, October 15, 2020. United States Department of Health and Human Services. National Institutes of Health. National Institute on Drug Abuse, and United States Department of Health and Human Services. Food and Drug Administration. Center for Tobacco Products. Population Assessment of Tobacco and Health (PATH) Study [United States] Restricted-Use Files. ICPSR 36231-v13. Ann Arbor, MI: Inter-university Consortium for Political and Social Research [distributor], November 5, 2019. Https://doi.org/10.3886/ICPSR36231.v23.

Table 6 reports the estimated hazards and its 95% CIs of age of initiation of each cigarillo outcomes by race/ethnicity. Non-Hispanic Black young adults exhibited a heightened risk for initiating ever use, past 30-day use and fairly regular use at earlier ages. By age 21, 11.7% of Non-Hispanic Black, 5.6% of Hispanic, 5.3% of Non-Hispanic White, and 2.0% of Non-Hispanic Other young adults initiated ever cigarillo use. By age 21, 9.8% of Non-Hispanic Black, 4.5% of Hispanic, 3.2% of Non-Hispanic White, and 1.2% of Non-Hispanic Other young adults initiated past 30-day cigarillo use. Finally, by age 21, 3.8% of Non-Hispanic Black, 1.4% of Hispanic, 1.2% of Non-Hispanic White, and 0.3% of Non-Hispanic Other young adults initiated fairly regular cigarillo use. Figs 1C, 2C and 3C also display the hazard functions of the ages of initiation for all three outcomes stratified by race/ethnicity.

**Table 6:** Estimated hazard functions^a^ (and 95% confidence intervals)^b^ of age of initiation of each cigarillo use outcome for the overall sample of PATH USA young adult (aged 18–24 at the first wave of participation) by race/ethnicity*.

Age	Non-Hispanic White	Hispanic	Non-Hispanic Black	Non-Hispanic Other^Ϯ^
**Ever Use of Cigarillos**				
**18**	0.0	0.0	0.0	0.0
**19**	2.1 (1.5–2.6)	1.9 (1.2–2.7)	3.5 (2.3–4.7)	1.1 (0.5–1.6)
**20**	3.8 (3.1–4.6)	3.9 (2.8–4.9)	8.8 (6.6–11.0)	1.6 (0.8–2.4)
**21**	5.3 (4.3–6.3)	5.6 (4.6–6.6)	11.7 (9.6–13.7)	2.0 (1.2–2.8)
**22**	6.7 (5.5–7.9)	7.4 (5.9–8.9)	15.2 (12.6–17.9)	2.3 (1.4–3.3)
**23**	7.4 (6.3–8.5)	9.0 (6.7–11.2)	17.4 (14.6–20.3)	2.3 (1.4–3.3)
**24**	9.5 (7.7–11.4)	11.3 (8.8–13.8)	21.9 (17.8–26.1)	3.9 (2.2–5.7)
**25**	11.8 (9.5–14.2)	14.6 (11.1–18.1)	28.2 (24.0–32.4)	6.0 (3.0–9.0)
**26**	13.3 (10.3–16.3)	19.0 (10.9–27.0)	30.7 (25.6–35.7)	6.0 (3.0–9.0)
**Past 30-day use of Cigarillos**				
**18**	0.0	0.0	0.0	0.0
**19**	1.1 (0.7–1.4)	1.6 (1.0–2.2)	2.6 (1.4–3.8)	0.5 (0.2–0.8)
**20**	2.3 (1.7–2.9)	3.0 (1.8–4.2)	7.1 (4.9–9.3)	0.9 (0.3–1.5)
**21**	3.2 (2.4–4.0)	4.5 (3.4–5.6)	9.8 (7.9–11.7)	1.2 (0.6–1.8)
**22**	4.1 (3.1–5.0)	6.0 (4.6–7.5)	13.3 (10.8–15.7)	1.5 (0.8–2.3)
**23**	4.6 (3.6–5.5)	7.6 (5.4–9.7)	15.0 (12.5–17.5)	1.5 (0.8–2.3)
**24**	6.0 (4.6–7.5)	9.2 (7.1–11.3)	18.6 (14.9–22.4)	2.1 (1.1–3.0)
**25**	6.7 (5.2–8.2)	11.1 (8.7–13.4)	23.9 (20.4–27.5)	3.1 (1.3–4.8)
**26**	8.4 (5.1–11.7)	13.5 (9.1–17.8)	25.7 (20.9–30.6)	3.1 (1.3–4.8)
**Fairly Regular Use of Cigarillos**				
**18**	0.0	0.0	0.0	0.0
**19**	0.4 (0.0–0.7)	0.3 (0.0–0.6)	1.0 (0.2–1.8)	0.0
**20**	0.8 (0.3–1.2)	0.3 (0.0–0.6)	3.3 (1.8–4.7)	0.0
**21**	1.2 (0.7–1.7)	1.4 (0.5–2.2)	3.8 (2.6–5.0)	0.3 (0.0–0.9)
**22**	1.2 (0.7–1.7)	1.8 (0.7–3.0)	4.9 (3.4–6.3)	0.3 (0.0–0.9)
**23**	1.4 (0.8–2.0)	2.3 (0.9–3.8)	6.1 (4.2–8.0)	0.3 (0.0–0.9)
**24**	1.4 (0.8–2.0)	2.6 (0.7–4.4)	8.0 (5.2–10.8)	1.7 (0.0–3.6)

^a^: Hazards are reported as cumulative percentages (i.e., cumulative incidence), which do not adjust for other covariates.

^b^: 95% CI: Turnbull 95% confidence interval.

*PATH Restricted file received disclosure to publish: July 15, 2020, October 15, 2020. United States Department of Health and Human Services. National Institutes of Health. National Institute on Drug Abuse, and United States Department of Health and Human Services. Food and Drug Administration. Center for Tobacco Products. Population Assessment of Tobacco and Health (PATH) Study [United States] Restricted-Use Files. ICPSR 36231-v13. Ann Arbor, MI: Inter-university Consortium for Political and Social Research [distributor], November 5, 2019. Https://doi.org/10.3886/ICPSR36231.v23.

^Ϯ^Non-Hispanic Others include Asian, multi-race, etc.

## Discussion

To our knowledge, our study is the first in the field to use survival analysis to estimate the distribution of the age of initiation of ever, past 30-day and fairly regular cigarillos use among young adults, between 2014 and 2017 who reported never cigarillo use at their first wave of adult participation (2013–2016) in the PATH study. By age 21, over 5% of young adults had initiated ever cigarillo use and 4% had initiated past 30-day use. Our data indicate that the estimated proportion of young adults who initiated past 30-day cigarillo use is more than doubled between ages 21 and 26. At the end of 2019, Tobacco 21 became law nationwide, rendering sales of tobacco products, including cigarillos, to anyone under the age of 21 illegal [35]. Future studies will be needed to examine if Tobacco 21 is effective in reducing underaged cigarillo and other tobacco product sales and use, as this law went into effect after our study period. While the impact of Tobacco 21 is promising, our data indicate that cigarillo ever, past 30-day and fairly regular use initiation persists beyond age 21 and that additional, sustained interventions and policy efforts are needed to curtail use among this group into early young adults.

Prior studies show that males and non-Hispanic Black young adults are at heightened risk for cigar product use [4–6]. Our data also show that young adult males, compared to females were more likely to initiate ever and past 30-day cigarillo use at younger ages. Compared to non-Hispanic Whites, non-Hispanic Blacks were more likely to initiate ever and fairly regular cigarillo use at earlier ages. Non-Hispanic Blacks and Hispanics were also more likely to initiate past 30-day cigarillo use at earlier ages than Non-Hispanic Whites. Self-identifying as non-Hispanic other appeared to be a protective factor for cigarillo initiation, as this group was less likely than non-Hispanic Whites to initiate ever and past 30-day cigarillo use at earlier ages. While identifying reasons for cigarillo use initiation is beyond the scope of this paper, evidence suggests that there is pervasive cigar product advertising in non-Hispanic Black and other communities of color and in young adult neighborhoods [36]. Cigarillo product advertisements targeted to young adults of color are also found online. For example, the official Swisher Sweets webpage (https://swishersweets.com/) includes several images of young adults, mostly male, who appear to be non-Hispanic Black and other ethnicities of color, giving the impression that these groups are representative of those who use and are attracted to the product. Content analysis of cigarillo mobile websites and social media accounts have documented the marketing tactics, such as using hip-hop and other influential cultural references, that are used by the industry to attract and appeal to young adults [37–39]. Tobacco product advertising and promotion exposure in young adults is associated with product initiation and continuation [40]. As noted previously, cigarillos are often used to smoke blunts [11–14]. Evidence suggests that use of cigarillo for blunt smoking is increasingly prevalent among non-Hispanic Black young adults [17, 41, 42]. The heightened risk of an earlier age of initiation of ever, past 30-day and fairly regular cigarillo use among non-Hispanic Black young adults may be due to blunt smoking. Moreover, our data indicate that young adults who had a history of marijuana or blunt use prior to cigarillo initiation were at heightened risk of an earlier age of initiation of ever, past 30-day, and fairly regular cigarillo use. Additional studies are needed to develop interventions that may reduce the transition from marijuana use to initiation of cigarillos during young adulthood. The targeted marketing of cigar products to young adults, particularly males and those who self-identify as people of color, use of cigarillos for blunt use, and with the lack of cigar prevention programs for young adults, may explain the heightened risk of initiation among this group.

Our study findings also highlight the risk of an earlier age of cigarillo initiation among those with other tobacco product use experience. Compared to non-tobacco product users, young adults with a history of cigarette and smokeless tobacco use were significantly more likely to initiate ever, past 30-day, and fairly regular cigarillo use at earlier ages than participants who did not use them after controlling for the other covariates. Filtered cigar users were more likely to initiate fairly regular cigarillo use at earlier ages than participants who did not use the products. However, those with a history of hookah use were less likely to report initiation of fairly regular cigarillo use at an earlier age than those who did not use hookah previously. Our findings are supported by other PATH study data that indicate that young adults with a history of previous use of other tobacco products were at increased risk of initiating past 30-day cigarillo use [22]. Although additional studies are needed to examine if young adults with a history of other tobacco product use are substituting with, switching to, or concurrently using cigarillo products, the use of multiple tobacco products among young adults is concerning. Prior studies suggest that cigar users who dual use with other tobacco products may be at increased risk of becoming addicted to nicotine and experience difficulty quitting smoking [43].

Our study has many strengths, including the use of a national (PATH) dataset, the precise measurement of the age of initiation across four waves of PATH data (within a week’s precision), use of the prospective design which avoids recall bias, and use of the interval-censored Cox proportional hazards models. Our study is not without limitations. PATH measures the cigarillo outcomes among users and non-users. We only included participants who were never users of cigarillos at their first wave of adult participation in PATH, leading to left-censoring subjects, which may lead to an overestimation of the age of initiation. However, our goal was to prospectively estimate the distribution of the age of initiation of cigarillo outcomes among never users of cigarillos who were aged 18–24 years old at their first wave of adult participation in PATH to study how these behaviors emerged over time among this age group. These never users of cigarillos did not initiate their use during adolescence and for that reason it is important that the public understand that young adults still initiate at later ages even if they did not initiate during their adolescent years. Another limitation, is the subjective measure of fairly regular use, as participants may interpret this question differently. Finally, we did not include other potential confounders, such as sociodemographic factors (e.g., household income, education level), intrapersonal factors (e.g., tobacco product knowledge, perception of harmfulness, perception of addictiveness, internalizing problems, externalizing problems), interpersonal (e.g., family history of tobacco product use, peer use of tobacco products), as well as environment factors (e.g., social media, advertisements, marketing). Investigating the impact of these risk factors on the age of initiation of cigarillo use will be important for future research.

In conclusion, our study documents the onset of cigarillo initiation among young adults–legal advertising targets for the tobacco industry. Our findings have important implications for intervention and tobacco control regulatory policy development. Most tobacco prevention interventions are developed for and implemented among adolescents, and primarily target cigarette or ENDS use. Our study findings highlight the importance of developing cigarillo-specific prevention interventions, including health communication campaigns, that address use among young adults and to educate the public domain. Prevention interventions should be targeted to young adults who identify as men, non-Hispanic Black and Hispanic, and have a history of marijuana/blunt, cigarette, and filtered cigar use as early as possible to reduce the initiation of cigarillo use.
